# Regulon Reconstruction Uncovers Novel Deregulated Factors in Alzheimer’s Disease

**DOI:** 10.1007/s12035-026-05745-8

**Published:** 2026-02-23

**Authors:** Marcella Vitória Belém-Souza, Gustavo Barra-Matos, Gilderlanio Santana de Araújo

**Affiliations:** 1 Graduate Program in Genetics and Molecular Biology, Institute of Biological Sciences, Federal University of Pará, Belém, 66075-100 Brazil; 2 Laboratory of Bioinformatics and Data Science, Institute of Biological Sciences, Federal University of Pará, Belém, 66075-100 Brazil

**Keywords:** Regulatory Transcriptional Network, Transcriptional Factors, Alzheimer's disease

## Abstract

Alzheimer’s disease (AD) is a progressive neurodegenerative age-related disorder characterized by widespread transcriptional deregulation across multiple brain regions. Among the molecular players involved, the transcription factors (TFs) can regulate the expression of AD-related peptides (β‐amyloid and tau). We aim to unveil reconstructed TF-centered networks and their dynamics across multiple brain regions. In this study, we conducted an exhaustive differential gene expression analysis, reconstructed TF-TF-centered regulatory networks, and performed master-regulation analyses across multiple regions. We used bulk RNA-seq data from 2,229 post-mortem samples from the ROSMAP, MAYO, and MSBB cohorts. To place these regulatory programs in a disease-relevant context, we integrated protein–protein interaction (PPI) data, experimental TF-target data, and AD-associated genetic risk loci as a translational layer. We assessed TF-centered regulons for 1,605 TFs and identified 354 master-regulators (MR-TFs) across multiple brain regions, including the parahippocampal gyrus, temporal cortex, and cerebellum, which exhibited the highest numbers of regulons. Overall, regulons fell within a moderate size range (median 55 targets), rather than into extensive large networks. Novel MR-TFs, including *ADCYAP1, TEAD2, BCL6, MAFF, NFKBIA,* were consistently identified as MR-TFs across tissues in AD. Furthermore, *GUCY1B1*, *RBFOX2*, and *MEF2C* were found conserved in the parahippocampal gyrus, inferior frontal gyrus, and posterior cingulate cortex*.* Additionally, our work identified the well-known AD-related genes *BIN1*, *EGFR*, and *SPI1* as MR-TFs, reinforcing their functional roles as susceptibility risk markers in AD. This work established an MR-TF-centered integrated regulatory network map of AD, revealing MR-TFs as factors that orchestrate gene deregulation in a region- and cell-context–dependent approach, and providing a robust foundation for mechanistic and translational investigations in neurodegeneration.

## Introduction

Alzheimer’s disease (AD) is a progressive neurodegenerative disorder influenced by genetic and environmental factors, and is the most common form of dementia [1, 2]. AD is marked by memory impairment, cognitive decline, and ultimately, impairment of activities of daily living. At the molecular level, AD is characterized by neuronal loss, synaptic dysfunction, and the accumulation of neuropathological lesions, including the hallmarks of amyloid-beta (Aβ) plaques and neurofibrillary tangles composed of hyperphosphorylated tau proteins [3]. The molecular heterogeneity underlying AD etiology continues to hinder comprehensive mechanistic understanding and the development of effective therapeutic interventions [4]. Large-scale transcriptome-wide and genome-wide association studies (GWAS) have robustly confirmed several well-established genetic associations with AD and have also identified novel susceptibility loci [1]. These discoveries have reshaped our understanding of AD pathophysiology, revealing layers of transcriptional and regulatory complexity that extend beyond the classical Aβ and tau-centric models [5].

To date, AD pathology has been extensively studied using bulk RNA sequencing (RNA-seq), primarily from peripheral blood samples. However, growing evidence highlights the spatial and cellular heterogeneity of transcriptional changes in the brain, with AD progression accompanied by the dynamic reprogramming of transcriptional regulatory networks at the cellular level across distinct brain regions, including the hippocampus, entorhinal cortex, and cingulate cortex. In this setting, region-specific molecular signatures suggest that AD unfolds within a heterogeneous landscape of transcriptional and cellular states [6–8].

In the context of transcriptional regulatory networks, recent research highlights transcription factors (TFs) as master modulators of AD-related gene expression, potentially reprogramming regulatory networks [9]. For example, *FOXP1* regulates *SORL1* expression, thereby promoting clearance of Aβ and amyloid precursor protein (APP), key mediators of AD pathology [10]. In contrast, *C/EBPβ* accelerates disease progression by modulating both inflammatory and cell-differentiation pathways [11]. TFs such as *Nrf2* exert neuroprotective functions by counteracting Aβ-induced toxicity [9, 12]. Besides, regulatory gene network analysis identified mechanisms involving *MEF2C* and *SPI1* that may influence AD, as master regulators of the inflammatory response in microglia [13].

Elucidating the heterogeneity of TF activity across brain regions and determining how these regulatory programs contribute to phenotypic diversity may provide critical insights into the molecular mechanisms underlying AD. In this study, we systematically investigate the interactions between master transcription factors (MR-TFs), defined as TFs that act as key regulatory drivers, and their corresponding target gene sets (regulons) across multiple brain regions [14]. We performed differential gene expression analyses, reconstructed TF-centered transcriptional regulatory networks from large-scale bulk RNA-seq datasets to identify MR-TFs and characterize their tissue-specific activity, intra-regional connectivity, and conservation across brain regions. Furthermore, we integrate these regulatory networks with protein–protein interaction data, TF-target interaction data (ChIP-Seq), AD-associated genetic risk loci, and assess cell-type specificity of MR-TFs.

## Methods and Materials

### Study Samples

We accessed RNA-seq data from three large cohorts, available on the Accelerating Medicines Partnership for Alzheimer’s Disease (AMP‐AD) working group, described below:

- The ROSMAP corresponds to two longitudinal clinical-pathological cohort studies of aging and Alzheimer's disease (AD) conducted by RUSH University Medical Center: the Religious Order Study (ROS) and the Memory and Aging Project (MAP) [15]. ROS focuses on phenotypes related to aging and Alzheimer's disease in Catholic orders. At the same time, MAP investigates the decline in cognitive and motor function and its association with AD risk. This cohort includes samples of RNA-seq data from post-mortem donors of five different tissues, including dorsolateral prefrontal cortex (AD = 308, CT = 148), frontal cortex (AD = 24, CT = 25), head of the caudate nucleus (AD = 178, CT = 95), posterior cingulate cortex (AD = 156, CT = 102), and temporal cortex (AD = 26, CT = 25), of which the temporal cortex and frontal cortex are not included. The ROSMAP samples (N = 987) are classified as the AD group according to the following criteria: BRAAK Score > 3, CERAD < 3, and CogDX (cognitive diagnosis of ‘no cognitive impairment’) = 4. Conversely, the CT group is defined by a BRAAK Score < 4, a CERAD > 2, and a CogDX = 1.
- The Mount Sinai/JJ Peters VA Medical Center Brain Bank (MSBB) cohort encompasses the full spectrum of cognitive and neuropathological disease severity, in the absence of detectable non-AD neuropathology, across more than 2,000 well-characterized human brains. This search comprehensively covers 920 post-mortem brains, including five specific brain regions [16]. Neuropathological evaluations for the Consortium were conducted on each sample to establish a Registry for Alzheimer's Disease (CERAD) protocol, which classified the samples into two groups: AD (CERAD 2) and healthy individuals (CERAD = 1). These samples of RNA-seq data from MSBB’s post-mortem donors are referents to tissues as the frontal pole (AD = 132, CT = 90), inferior frontal gyrus (AD = 132, CT = 91), parahippocampal gyrus (AD = 146, CT = 83), prefrontal cortex (AD = 8, CT = 3), and superior temporal gyrus (AD = 149, CT = 86).
- The Mayo Clinic Study of Aging (Mayo) aims to compare the reconstruction of tissue-specific regulatory networks. The Mayo is a prospective, population-based cohort study in which in-person clinical evaluations are conducted at the Mayo Clinic Abigail Van Buren Alzheimer’s Disease Research Clinic or at participants’ residences, using standardized protocols. The Mayo Clinic study aims to investigate the prevalence, incidence, and risk factors of mild cognitive impairment and dementia [17]. This cohort comprises samples ranging in age from 60 to 90 years, including individuals with AD (84 in cerebellum, 82 in temporal cortex) and healthy individuals (78 in both cerebellum and temporal cortex), derived from post-mortem RNA-seq donor tissue data (N = 322 samples). The study relies on the NINCDS-ADRDA criteria to confirm a diagnosis of AD.

### Differential Gene Expression Analysis

Differential gene expression (DGE) analysis was performed between the CT and AD groups for each tissue using the *edgeR* package in R (version 4.4.1). *edgeR* examines differential expression in replicated read-count data for genes or genomic features, employing exact statistical methods and linear regression models [18]. To compare the CT and AD groups, sex and age were included as covariates. DGE results were considered statistically significant considering the threshold of fold-change |FC|> 0.5 and False Discovery Rate (FDR p-value ≤ 0.05). Gene ontology analysis was performed using the ClusterProfiler package in R (version 4.4.1) [19]. Biotype class and official gene symbols were mapped for each Ensembl transcript identifier using EnsDb.Hsapiens.v86 dataset (10.18129/B9.bioc.EnsDb.Hsapiens.v86).

### Reconstruction of Transcriptional Regulatory Networks

Reconstruction of Transcriptional Regulatory Networks was performed using the RTN package to identify TF-regulons [20]. This package employs the ARACNe (Algorithm for the Reconstruction of Accurate Cellular Networks) method to infer regulons. Significant TF-target interactions constitute the regulon. Mutual Information (MI) and permutation analysis are used to identify unstable TF-target interactions, which are further removed via bootstrap analysis.

Weak TF-triplet interactions (TF-TF-Gene) are removed by the Data Processing Inequality (DPI) filter. Here, the number of permutations, the number of bootstraps, and the p-value cutoff were set to 1000, 100, and 0.01, respectively. The activity mode is assigned to each TF-target based on MI and Spearman’s rank correlation, with positive values indicating activation and negative values indicating repression.

Master regulator analysis (MRA) was used to identify MR-TFs integrating with DGE results. The MRA was performed with a p-value cutoff of 0.05 using the Benjamini-Hochberg (BH)–adjusted p-value method, with a minimum of 15 targets per regulon. All parametrizations were based on Castro M. et al. [21].

### Data Integration

#### Transcription Factor and Target Interaction Data

To explore the TF network, we integrated a knowledge-based network using data from TFLink (version 1.0, accessed November 21, 2024) (https://tflink.net/). TFLink is a comprehensive repository that catalogs TF-target data, nucleotide sequences, and genomic coordinates across humans and other species [22]. From TFLink, we integrated data from small- and large-scale experimental studies, identifying 1,605 TFs that interact with 20,139 target genes across 6,739,357 catalogued interactions. TFLink integrates 10 major transcription factor databases, including DoRothEA, GTRD, HTRIdb, and JASPAR.

#### Protein–Protein Interaction Data for Alzheimer’s Disease (AD-PPI)

BioGRID curators collaborated with AD experts to develop a specialized AD-related protein–protein interaction network (AD-PPI). The curated AD-PPI is involved in Aβ production and tau protein modifications, including phosphorylation, ubiquitination, aggregation, folding, cleavage, and clearance [23]. The molecular interactions for these AD-related genes were manually curated (https://thebiogrid.org/project/7/alzheimers-disease.html, v.5.0.252, December 05, 2025) [24]. The AD-PPI comprises 193,265 interactions among 466 catalogued genes in AD.

#### Genome-Wide Association Study Signals

To assess functional convergence between MR-TFs and AD-related genes identified in genome-wide association studies (GWAS), we integrated genetic susceptibility summary statistics for AD from the up-to-date large GWAS [1], which tests SNP genotype data from 789,009 individuals (111,326 clinically diagnosed AD cases and 677,663 controls) and identified 75 risk loci, of which 42 were novel risk SNPs.

## Network Overlap Coefficient Analysis

We used the overlap coefficient (Szymkiewicz–Simpson coefficient) for two complementary purposes: (i) to quantify the similarity between brain tissues based on DGE results, and (ii) to assess the conservation of transcriptional regulation at the level of TF–target interactions across tissues.

Let *T*_*1*_ and *T*_*2*_ denote the sets of DGE results in pairs of tissues, respectively. The overlap coefficient between the two tissues is defined as:

$$\text{Overlap Coeficient }\left({T}_{1}, {T}_{2}\right)= \frac{| {T}_{1}\cap {T}_{2}|}{\mathrm{min}\left(|{T}_{1}|, \right|{T}_{2}|)}$$

This formulation quantifies the proportion of shared transcripts relative to the smaller transcript set, enabling direct comparison between tissues with different numbers of differentially expressed transcripts.

To assess the conservation of regulon topology across brain tissues, we quantified network overlap using the same metric. Let *R*_*1*_ and *R*_*2*_ represent the regulons (MR-TF-target edges) inferred for the same MR-TF in distinct tissues, defined as:

$$\text{Overlap Coeficient }\left({R}_{1},{R}_{2}\right)= \frac{| {R}_{1}\cap {R}_{2}|}{\mathrm{min}\left(|{R}_{1}|, \right|{R}_{2}|)}$$

The overlap coefficient ranges from 0 to 1. Higher values indicate greater overlap between the two sets, with 1 indicating that the smaller set is entirely contained within the larger set.

## TF-Centered Single-Cell Analysis

The Cell Marker 2.0 database [25] provides a manually curated resource of cell-type–specific markers, including genes and TFs, associated with different cell types, tissues, and clinical conditions. Cell markers are derived predominantly from published experimental evidence, such as scRNA-seq, snRNA-seq, transcriptomic analyses, and immunohistochemical experiments. In this study, we used data derived from human samples, which initially comprised 12,937 marker genes mapped with 1,715 distinct cell types. These data were subsequently filtered by tissue type, retaining only entries corresponding to brain tissue, yielding a dataset comprising 206 cell types and 1,448 marker genes. The filtered dataset consists of a tabulated mapping of cell types to their associated molecular markers, in which each entry links a marker to the cell population, the tissue of origin, and the corresponding experimental evidence. The brain cell markers were then compared with the MR-TF list, enabling the identification and annotation of cell-type transcriptional signatures across distinct brain cell populations.

## Results

### Transcript Deregulation Across Brain Regions

**Fig. 1:**
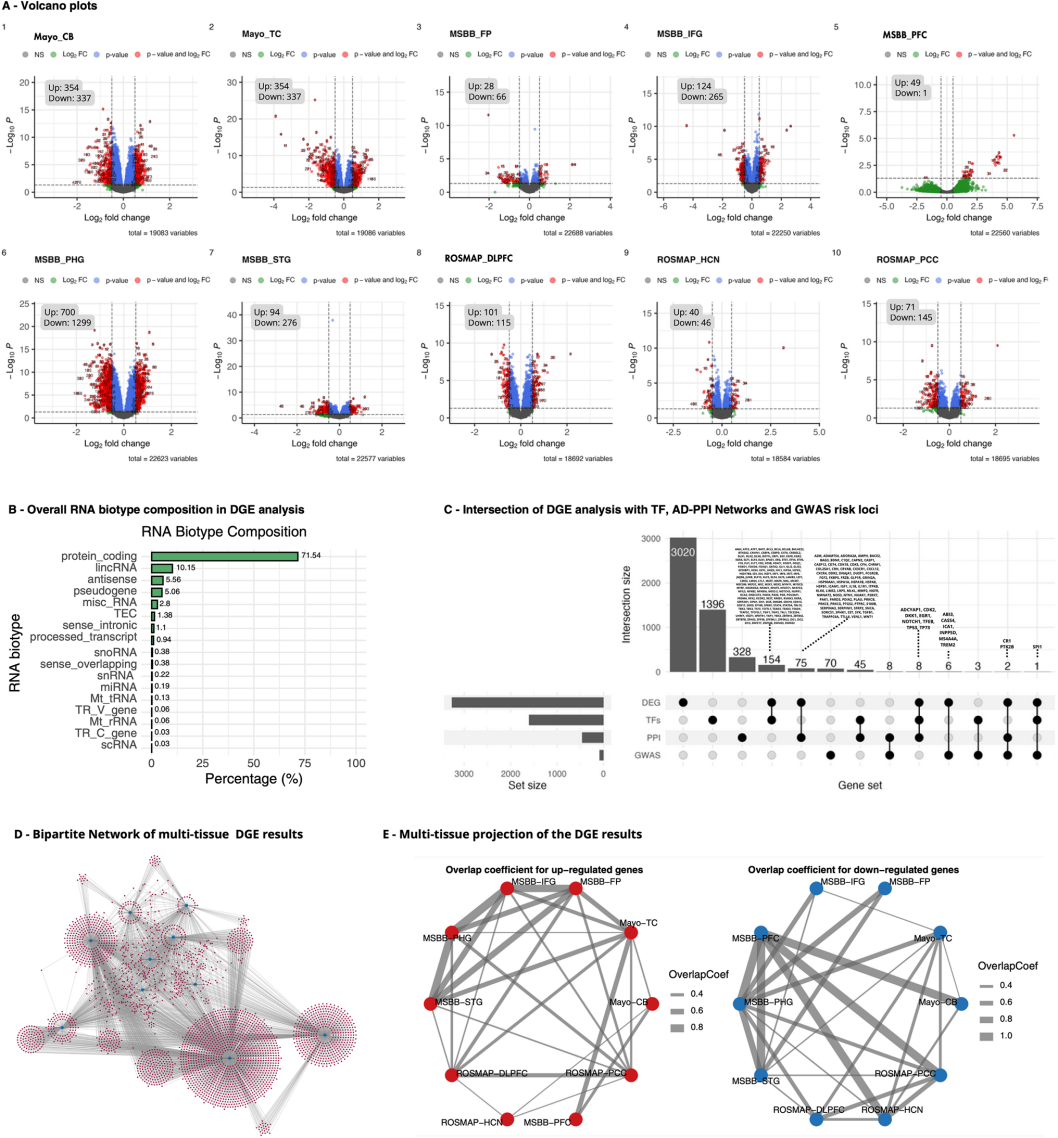
Differential Gene Expression results and brain-tissue connectivity. **A**) (A1-A10) Volcano plots by brain regions. Red dots indicate differentially expressed genes (FDR ≤ 0.05 and |logFC|> 0.5). **B**) Biotype distribution of transcripts in DGE results. **C**) Overlap between gene sets in DGE results, AD-associated genes in Bellenguez et al. (2022) GWAS, AD Protein–Protein Interaction Network (AD-PPI, BioGrid), and TF-target interaction data (TFLink database). **D**) Bipartite network projection of the DGE analysis, illustrating the relationships between genes (red dots) and tissues (blue dots). **E**) Weighted projection of the bipartite network using the overlap coefficient index to quantify pairwise gene set similarity for up-regulated and down-regulated genes. The width of each edge is proportional to the overlap coefficient between two tissues. DGE: differential gene expression; CB: Cerebellum (MAYO); TC: Temporal Cortex (MAYO); FP: Frontal Pole (MSBB); IFG: Inferior Frontal Gyrus (MSBB); PFC: Pre Frontal Cortex (MSBB); PHG: Parahippocampal Gyrus (MSBB); STG: Superior Temporal (MSBB); DLPFC: Dorsolateral Prefrontal Cortex; HCN: Head of the Caudate Nucleus (ROSMAP); PCC: Posterior Cingulate Cortex (ROSMAP)

Differential gene expression was assessed for all cohorts and brain tissues using a statistical threshold of |FC|> 0.5 and FDR ≤ 0.05. In the multi-tissue analysis, a total of 3,266 transcripts were identified as differentially expressed (Fig. 1. A.1–A.10). DGE results revealed widespread and region-specific transcriptional deregulation in AD. Regulatory deregulation affected multiple cortical and subcortical regions, including the temporal cortex, cerebellum, inferior frontal gyrus, superior temporal gyrus, dorsolateral prefrontal cortex, posterior cingulate cortex, and frontal pole (Fig. 1A), with a consistent predominance of downregulated transcripts. The parahippocampal gyrus, an early-affected region, exhibited the most extensive changes, with 700 upregulated and 1,299 downregulated transcripts (Fig. 1-A.6). In contrast, the prefrontal cortex displayed a marked enrichment of upregulated genes.

Gene biotype annotation revealed a strong predominance of protein-coding genes, which accounted for 71.53% of the analyzed dataset (2,277 genes). Among non-coding transcripts, lincRNAs (10.14%) and antisense RNAs (5.56%) were the most abundant classes, followed by pseudogenes (5.05%) and miscellaneous RNAs (2.7%). All other biotypes, including sense intronic, sense overlapping, snoRNAs, snRNAs, miRNAs, and mitochondrial RNAs, individually contributed less than 5.02% of the total (Fig. 1B).

Data integration analysis revealed functional convergence across DGE results, genetic risk mapping, TF-target networks, and AD-PPI network (Fig. 1C). TF *SPI1* was identified as a deregulated focal element in which genetic susceptibility, transcriptional regulation, and expression changes converge. Our mapping analysis integrating DGE results, AD-PPI, and TF-target interaction data identified eight genes (*ADCYAP1*, *CDK2*, *TFEB*, *TP73*, *EGR1*, *NOTCH1*, *TP53*, and *DKK1*). The intersection between DGE results, GWAS signals, and PPIs highlights *CR1* and *PTK2B*, both of which are well-recognized genetic risk factors for AD. The intersection between DGE results and GWAS loci comprised six genes (*ABI3, TREM2, CASS4, MS4A4A, ICA1,* and *INPP5D*). This overlap indicates that a subset of TFs directly maps onto inherited AD-risk variants, strengthening the functional relevance of these genes. Beyond these overlaps, an additional set of 154 TFs showed differential expression, reflecting widespread transcriptional reprogramming in AD. The overlap between DGE results and GWAS loci (six genes) and between DGE results and AD-PPIs (75 genes) suggests intermediate levels of functional support, linking expression changes either to inherited risk or to downstream protein networks.

We modeled a bipartite network where nodes are two disjoint sets: tissues and genes with differential expression (Fig. 1D). Subsequently, based on the bipartite network, we constructed two tissue-centered projections, one for up-regulated and one for down-regulated elements, using the overlap coefficient as the similarity measure (Fig. 1E). Our analysis revealed heterogeneity in transcriptional signatures across independent cohorts and brain regions. Consistency within the MSBB study was notably high, with the inferior frontal gyrus and parahippocampal gyrus axis exhibiting the strongest concordance in up-regulated genes (overlap coefficient 0.83). However, the robustness of these signatures declined significantly in cross-cohort comparisons. For instance, the overlap between Mayo-temporal cortex and MSBB cortical regions ranged from 0.44 to 0.53. In contrast, the cerebellum exhibited a distinct molecular profile, with minimal overlap (typically < 0.10) with cortical areas. While upregulated genes showed strong intra-study regional cohesion, downregulated signatures exhibited superior preservation across disparate cohorts. Notably, the overlap of downregulated genes between parahippocampal gyrus and ROSMAP-dorsolateral prefrontal cortex (0.53) substantially exceeded the overlap observed for upregulated genes (0.34) between the same regions.

## Reconstructed Transcription Factor Networks in Alzheimer’s Disease

We reconstructed TF-centered regulatory networks across ten brain regions and retained regulons driven by master regulators (MR-TFs; adjusted *p* < 0.05; Table 1). Table 1 summarizes the total number of inferred regulons, the number of MR-TFs, and their direction of regulation across brain regions. For each brain tissue, Table 1 further distinguishes MR-TFs with upregulated and downregulated expression, highlighting region-specific regulatory profiles.

**Table 1:** Summary of the total of inferred TF regulons by tissue, including the number of MR-TF regulons and their deregulation (up- and down-regulated)

Tissue	#Regulons	#MR-TFs	Upregulated MR-TFs	Downregulated MR-TFs
DLPFC	1035	14	*ADCYAP1*	*FOXO4, TEAD2, GLI1, MAFF*
HCN	996	18	*BATF*	*GLI1*
PCC	967	22	*-*	*FOXO4, GLI1, ID3, MAFF, MCM7, MSX1, NUPR1, SERTAD1, TEAD2*
CB	1153	90	*HSD17B8, RCOR2, TFCP2L1*	*JMJD6, MAFF, YBX3*
TC	1109	113	*ADCYAP1, DLX1, DLX6, EGR1, GPIHBP1, MAL, POU2AF1*	*CDK2, EGF, ETV4, F10, FLT1, FOXC1, GLI1, GLIS3, ID3, ID4, KLF2, KLF5, MECOM, MSX1, NFKBIA, PAX6, PGR, PRDM6, SMAD6, TBX3, TEAD2, WWTR1, YAP1, ZIC1*
IFG	975	59	*ADCYAP1, EGR4*	*BCL6B, FOXC1, HIF3A, MECOM, TBX3*
STG	996	38	*ADCYAP1*	*FLI1, MECOM, TEAD2*
FP	1038	14	*ADCYAP1*	*-*
PFC	233	0	*-*	*-*
PHG	982	167	*ADCYAP1, LMO4, MEF2C, MSC, OCA2, STAT4*	*ATF7, BCL3, BCL6, BTN3A2, CAVIN1, CEBPA, CEBPD, CIITA, CREB3L2, EBF1, ELF1, ELF4, EPAS1, ETS1, FLI1, FLT1, FOXC1, FOXJ1, HIF3A, HIPK2, ID3, IKZF1, IRF7, KLF15, LEF1, MAFF, MECOM, MYH11, MYRF, NFKB2, NOTCH1, NOTCH3, PGR, REST, RFX2, RREB1, RXRA, SIPA1, SOX10, SOX13, SOX9, SPI1, STAT5A, TBL1X, TBX3, TFEB, TGIF1, VEZF1, WWTR1, YAP1, YBX3, ZBTB7B, ZFHX3, ZFP36, ZFP36L1, ZIC2, ZNF423, ZNF652*

**Fig. 2:**
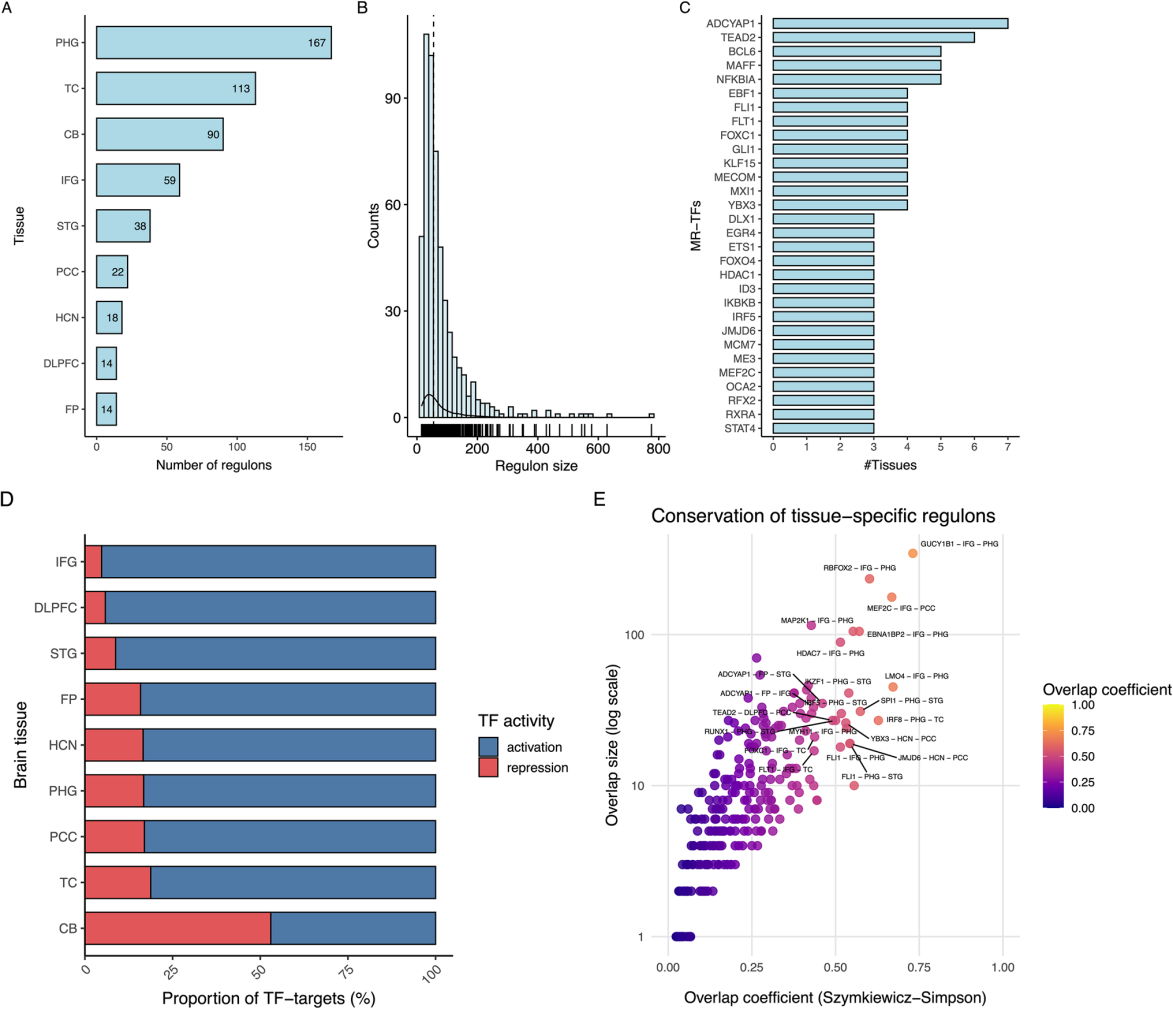
Descriptive statistics of MR-TF regulons. **A**) Counts of MR-TFs by brain region. **B**) Distribution of regulon size by MR-TFs. **C**) Top-30 shared MR-TFs across brain regions. **D**) Proportion of MR-TF-target activity across brain regions, showing activation (blue bar) and repression (red bar). **E**) Regulatory conservation (overlap coefficient) and tissue specificity in view of MR-TF-target interaction sets. Yellow dots indicate higher regulatory conservation for the pair, and blue dots indicate lower regulatory conservation

Although a large number of regulons were initially inferred, statistical filtering substantially reduced this number. Across all tissues, the number of regulons is relatively similar, ranging from approximately 950 to 1,150, indicating a comparable level of overall regulatory complexity. However, the number of MR-TFs varies substantially across tissues, reflecting different degrees of reorganization or specific transcriptional control. In total, we assessed regulons for 1,605 TFs and detected 354 MR-TFs in multiple tissues. Among the brain regions examined, the parahippocampal gyrus showed the highest concentration of MR-TFs, with 167 detected, distinguishing itself with the most extensive array of both activated and repressed factors. Following the parahippocampal gyrus were the temporal cortex (n = 113) and the cerebellum (n = 90) (Fig. 2A). The distribution of regulon size revealed substantial variability, ranging from small regulons (~ 15 target genes) to very large (~ 700 target genes) per MR-TF. Most regulons fell within a moderate size range (median 55 target genes) (Fig. 2B), indicating that MR-TF control focused on transcription programs rather than on extensive networks.

We observed several MR-TFs across multiple brain tissues (Fig. 2C). A restricted subset of MR-TFs exhibited cross-regional recurrence, including upregulation of *ADCYAP1* (7 tissues), downregulation of *TEAD2* (6 tissues), and downregulation of *BCL6*, *MAFF*, and *NFKBIA* (each detected in 5 tissues), suggesting core regulatory programs shared across brain areas.

In relation to MR-TF control, we observed a predominance of activation over repression in MR-TF regulons across multiple tissues, except in the cerebellum (Fig. 2D). Of particular note, the inferior frontal gyrus and dorsolateral prefrontal cortex showed higher control activation (> 75%) than the cerebellum, where activation and repression were similar (~ 50%).

We performed pairwise overlap comparisons of interactions from MR-TF regulons across multiple brain regions to quantify regulatory conservation and tissue specificity (Fig. 2E). Overall, most MR-TF regulatory regions exhibited low to moderate overlap across tissues, indicating substantial regional specificity in transcriptional regulation. This pattern was particularly evident in comparisons involving the cerebellum, which frequently showed low overlap coefficients (e.g., *PGBD5, ZNF554, RCOR2, IKBKB*). In contrast, a subset of TFs displayed highly conserved regulons across cortical areas, characterized by high overlap coefficients. Notable instances include *GUCY1B1, RBFOX2, MEF2C, LMO4, HDAC7, IRF8,* and *IKZF1*, which showed overlap coefficients exceeding 0.5 in several tissue pair comparisons (Fig. 2E).

Directional containment analysis revealed asymmetric regulatory relationships, in which smaller MR-TF regulons were frequently nested within larger ones. *GUCY1B1* and *ADCYAP1* represent two contrasting cross-tissue regulatory architectures. *GUCY1B1* exhibits extensive conservation of TF–target interactions across cortical regions, with numerous shared edges and high overlap coefficients, indicating a stable, tissue-invariant core regulon. In contrast, *ADCYAP1* was active across seven tissues, was of moderate size, and exhibited a moderate overlap coefficient.

## Data Integration Supported by PPI, TF-Target, and GWAS Risk Alleles

Inferred MR–TF regulons were mapped to AD-PPI data to assess the extent of physical interactions supported. Table 2 lists the MR–TF regulons ranked by the number of AD-PPI–supported interactions. Across tissues, a subset of MR–TF-targets showed evidence of AD-PPIs, typically 1–6 interactions, despite substantial variation in regulon size (27–629 targets). MR–TFs such as *CDK2* and *CSNK2A1* in the temporal cortex exhibited the highest number of supported experimental interactions. Several MR–TFs (e.g., *CSNK2A1, MAP2K1, YBX3, KAT7*) were detected across multiple tissues but often interacted with distinct partners, showing tissue-specific regulatory wiring. Notably, many supported interactions involved proteins with roles in neurodegeneration and AD pathology, including *APP, SNCA, PRNP, PINK1, CASP1/4*, components of the *NOTCH* pathway, and *TP53*-associated factors. These results indicate that although MR–TF regulons can be extensive, only a small, high-confidence fraction is corroborated by AD-relevant PPI evidence.

**Table 2:** Summary of master regulator transcription factors (MR-TFs) identified across multiple brain tissues and their physical interactions from an AD protein–protein interaction (AD-PPI) network (BioGRID). AD-PPI data is generated by mass spectrometry and/or western blot methods. Tissue; MR–TF: TF as master regulators, Size: regulon size, Interactions: number of supported interactions; *: GWAS AD-related gene

#	Tissue	MR-TF	Size	Interactions		Targets on AD-PPI
1	TC	*CDK2*	43		6	*CASP4; CLIC1; IL15RA; PML; TAGLN2; YBX3*
2	TC	*CSNK2A1*	80		5	*ECD; KAT7; NAF1; NR2C2; PAFAH1B1*
3	FP	*CSNK2A1*	56		3	*HECW2; KPNA1; NR2C2*
4	CB	*NFE2L2*	154		3	*RIT1; SMARCA4; USP11*
5	IFG	*USP7*	190		2	*PPP2CA; SNCA*
6	PHG	*TP53BP1*	233		2	*COPS5; OGT*
7	PCC	*YBX3*	49		2	*YBX3; ICAM1*
8	PHG	*NOTCH1*	65		2	*NOTCH3; USP15*
9	IFG	*MAP2K1*	545		1	*HSP90AB1*
10	IFG	*LMO4*	629		1	*GSK3B*
11	CB	*ELK1*	93		1	*DNAJA1*
12	PHG	*MAP2K1*	190		1	*HSP90AB1*
13	IFG	*EAF1*	153		1	APP*
14	IFG	*XRCC3*	230		1	APP*
15	TC	*STAT3*	93		1	*FYN*
16	IFG	*MAML1*	394		1	*EP300*
17	PHG	*SVIL*	90		1	*PRNP*
18	CB	*JMJD6*	318		1	*HSPH1*
19	STG	*SCAND1*	160		1	*RER1*
20	CB	*SND1*	168		1	*PINK1*
21	IFG	*YAP1*	73		1	*OTUB1*
22	TC	*KAT7*	60		1	*CSNK2A1*
23	TC	*MYCN*	66		1	*TBC1D10B*
24	FP	*NR2C2*	96		1	*CSNK2A1*
25	CB	*YBX3*	58		1	*CDK2*
26	PCC	*KAT7*	68		1	*KAT7*
27	HCN	*YBX3*	53		1	*CDK2*
28	PHG	*RELA*	27		1	*ZBED6*
29	PHG	*YBX3*	36		1	*CDK2*
30	TC	*MXI1*	38		1	*CALCOCO2*
31	PHG	JUNB*	29		1	*JUN*
32	PHG	*NOTCH3*	36		1	*NOTCH1*
33	TC	*CEBPG*	60		1	*ATF4*
34	TC	BIN1*	69		1	*LIG1*
35	PHG	*IFI16*	31		1	*CASP1*

As part of an integrative analysis, we integrated MR-TF regulons with summary statistics for AD-risk loci. Table 3 summarizes the MR-TFs, the tissue in which the regulon was reconstructed, the regulon size, and the interactions with AD-risk loci. MR-TF regulons were mapped with GWAS signals in the parahippocampal gyrus, superior temporal gyrus, and temporal cortex. Overall, direct mapping of MR-TF regulons with GWAS results was sparse, highlighting evidence for *SPI1*, *EGFR*, and *BIN1*. Regulons for the MR-TF *SPI1* were identified in the PGH, targeting *ABI3* and *CTSB*, whereas in the STG, *SPI1* also targets *INPP5D*. Additionally, *EGFR* and *BIN1* were identified as MR-TFs located within established AD risk loci in the temporal cortex. However, their downstream regulon targets did not overlap with AD-related genes reported by Bellenguez et al. (2022) [1].

**Table 3:** Summary of master regulator transcription factors (MR-TFs) identified across multiple brain tissues and their interactions with AD-associated genome-wide association studies (GWAS) from Bellenguez et al. 2022. Tissue; MR–TF: TFs as master regulators; Size: regulon size, Interactions: number of supported interactions and interacting targets

Tissue	MR-TF	Size	#interactions	Target on GWAS’s summary
PHG	*SPI1*	54	2	*ABI3, CTSB*
STG	*SPI1*	81	2	*ABI3*, *INPP5D*
TC	*EGFR*	31	0	-
TC	*BIN1*	69	0	-

## MR-TF-Targets Mapping with TFLink

**Fig. 3:**
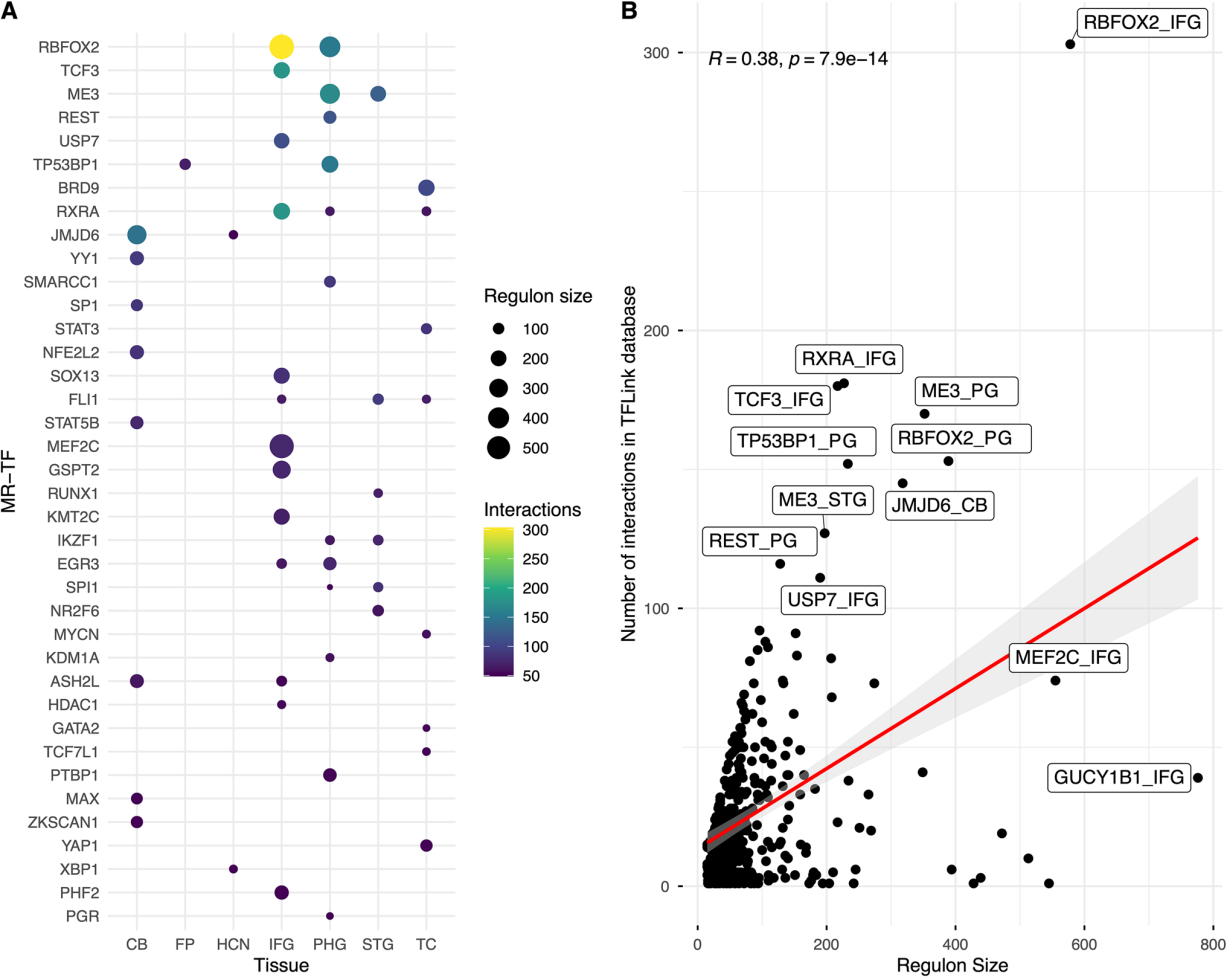
Mapping results of MR-TFs regulons with TF-target data from the TFLink database. **A**) Top 50 master regulator transcription factors (MR-TFs) identified across multiple brain tissues with the highest number of TF-target interactions shared with TFLink. Dot size represents the regulon size by brain regions, and the gradient color represents the number of TF-target interactions in TFLink for each regulon. **B**) Scatter plot and Spearman correlation test between the MR-TFs regulon size and the number of interactions supported in the TFLink database

Finally, we mapped the MR-TF regulons to the TFLink interaction data. These include evidence derived from both small- and large-scale experimental studies and from chromatin immunoprecipitation sequencing (ChIP-seq). Regulons for the MR-TFs *RBFOX2, RXRA, TCF3, ME3,* and *TP53BP1* showed the highest number of shared interactions with TFLink. The Top 50 MR-TFs and the distributions of regulon size and interaction count are shown in Fig. 3. These MR-TFs were predominantly observed in the parahippocampal gyrus, inferior frontal gyrus, and superior temporal gyrus. Among these, *RBFOX2* displayed the highest number of shared interactions with TFLink in the inferior frontal gyrus and parahippocampal gyrus, with more than 50% of its inferred regulon (52%) supported by previously validated TF–target interactions (Fig. 3A).

Regarding the distribution of interactions across MR-TF regulons, we observed a moderate positive correlation (Spearman’s r = 0.38, p < 0.05) between the number of TF–target interactions and the size of the regulon (Fig. 3B). This finding suggests that these regulatory networks can be partially reconstructed using validated interactions within specific regions; for instance, the MR-TFs *RBFOX2* (found in inferior frontal gyrus and parahippocampal gyrus) showed a high overlap of TF-target interactions with TFLink data.

## Single-Cell Analysis

**Fig. 4:**
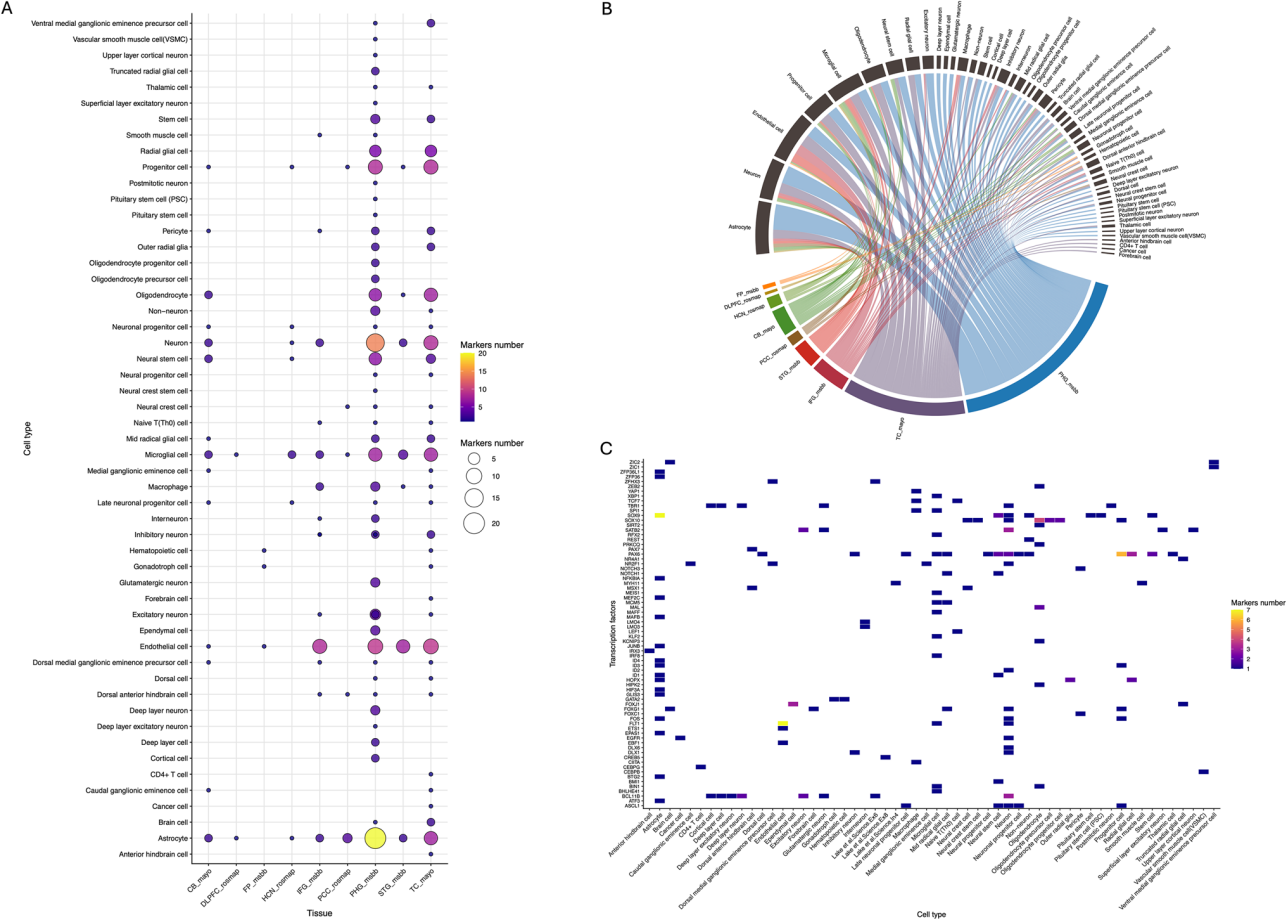
Master regulator transcription factors (MR-TFs) and single-cell analysis. **A**) MR-TFs identified in each brain tissue and their quantitative distribution across several cell types. **B**) Alluvial (flux) plot illustrates the distribution of MR-TFs across brain regions and their enrichment in specific cell types. **C**) MR-TFs identified across multiple brain tissues and their presence across different cell populations

We observed that the parahippocampal gyrus and temporal cortex tissues exhibited greater cellular diversity, as evidenced by a higher number of possible MR-TFs associated with astrocytes, endothelial cells, microglia, neurons, oligodendrocytes, progenitor cells, and radial glial cells (Figs. 4A and 4B). Among astrocytes, *SOX9* was the most frequently identified MR-TF, whereas in endothelial cells, the highest TF abundance was observed for *FLT1*. In the neuronal group, *BCL11B* and *SATB2* were the most prevalent TFs, whereas *SOX10* predominantly characterized oligodendrocytes. Progenitor cells showed *PAX6* as the most recurrent MR-TF, and radial glial cells were mainly marked by *PAX6* and *HOPX* (Fig. 4C).

## NeuroRegulonDB: a Statistical Dashboard for DEGs and MR-TF Analysis

Due to the number of analyses performed in this study, extensive statistical summary tables were generated as supplementary material. Thus, to facilitate data exploration, transparency, and reproducibility, we construct an online statistical dashboard as an interactive web-based platform that enables detailed inspection of the analyses. All results are publicly available through the NeuroRegulonDB application at https://lbcd.ufpa.br/neuroregdb.

The platform provides access to tabulated summary data for the following analyses:

- Differential gene expression (DGE) results;
- Gene ontology enrichment and term overlap analysis per tissue for DGE results;
- Regulons and master regulons (MR-TF) inferred for each tissue (raw data and network projection);
- Supporting interaction data based on integration with AD-PPI and TFLink.

## Discussion

Comprehensive transcriptomic profiling of AD revealed pervasive transcriptional deregulation across gene regulatory networks. Across multiple brain regions, we identified extensive reorganization of TF-centered regulons. Rather than being uniform or static, transcriptional deregulation reflects a dynamic, regionally structured regulatory architecture that may contribute to neurodegeneration. We performed an integrative analysis of differential gene expression and MR-TF-centered regulons across multiple brain tissues to reconstruct AD-associated regulatory gene networks. These analyses also led to the development of NeuroRegulonDB, an online statistical dashboard and resource that catalogues gene deregulation and inferred MR-TF regulons, and is integrated with AD-PPI and TF–target interaction databases.

Integrated analysis of multiple brain regions revealed that gene-expression deregulation in AD varies across anatomical contexts [6]. The parahippocampal gyrus exhibited the highest burden, with transcriptomic deregulation occurring first in the temporal cortex. These results reinforce that AD pathology does not manifest uniformly across the brain, but emerges from regional trajectories of molecular and cellular dysfunction [6, 26]. Furthermore, the transcriptional profile reflects disease progression, with early-affected areas (such as the parahippocampal and entorhinal zones) more vulnerable to transcriptional changes than later-affected areas, including the temporo-parietal cortex [6, 27].

The transcriptional direction of genes is a critical determinant of AD. Downregulated genes showed greater prevalence across regions and cohorts, suggesting that the loss of pathways regulating neuronal and synaptic function constitutes a pathological core of AD. Through analysis of deregulated genes, we observed enrichments for processes related to responses to mechanical stimuli, chemotaxis, decreased oxygen levels, and protein folding, which processes were shared across regions. Besides, we observed deregulated genes associated with risk for AD, such as *ABI3, TREM2, CR1,* and *PTK2B,* genes related to neuroinflammation and glial activation, including the involvement of the microglial immune response to Aβ and tau aggregates [1, 28–30].

Several MR-TFs may shape the neurodegeneration process. For example, Vargas et al. identified novel TFs *CNOT7, CSRNP2, SLC30A9*, and *TSC22D1*, as well as TFs already reported in AD, such as *ATF2* and *PARK2;* among them, 7 MR-TFs (*RRB1, HIVEP2, KDM1A, TAF7, YY1, TRIM2*8, and *KAT7*) were identified in common with our MRA [14]. A hippocampal neuron model of transcriptional regulation for AD was applied to identify potential MR-TFs across multiple tissues, thereby highlighting three novel TFs (*YY1, p300,* and *ZMYM3*) implicated in AD progression and neurodegenerative processes [31], however, among all MR-TFs identified, MR-TFS such *as BCL6, MAFF, TEAD2, SOX9, TCF3* and *NOTCH1* showed in common with our results.

Building on these approaches, our large-scale study to reconstruct regulatory networks identified hundreds of TFs that act as MR-TFs in AD. Among the MR-TFs we identified, *ADCYAP1, TEAD2, BCL6, MAFF,* and *NFKBIA* were detected across multiple regions. The recurrence of MR-TFs across multiple tissues reinforces the conception of a core regulatory mechanism involved in cellular processes that may be common across tissues and may be potential candidates for AD etiology. These MR-TFs coordinate regulons of moderated size, suggesting control of specialized functional programs rather than ubiquitous effects on extensive regulatory networks in AD. Consistent with previous reports, we found that *BCL6* is deregulated MR-TFs across several brain regions [32]. *BCL6* may influence AD by modulating the *NOTCH* signaling pathway, which plays a central role in brain development, neuronal differentiation, and neural stem cell maintenance, with established links to neurodegeneration [33, 34].

The MR-TF *ADCYAP1* (adenylate cyclase-associated protein 1) gene encodes the neuropeptide PACAP (Pituitary Adenylate Cyclase-Activating Polypeptide), which is involved in hormonal regulation, stress, neuroendocrine communication, and neurotransmission modulation, and protects against Aβ toxicity. Deregulation of *ADCYAP1* is associated with hippocampal alterations, with cognition, learning, and memory [35, 36]. *ADCYAP1* and *MAFF* were also identified as diagnostic markers for AD in an in silico study that integrated bioinformatics analyses with machine-learning strategies [37]. This positions both genes as statistically informative features that can support distinguishing AD cases from controls in the analyzed expression datasets, thereby identifying candidate diagnostic biomarkers [37, 38].

As TF repressors, *BLC6* and *MAFF* were shown to be MR-TFs linked to AD. The B cell Lymphoma 6 (*BLC6)* is a transcriptional repressor expressed in the human brain and involved in neuronal development. *BLC6* has also been linked to progressive AD by suppressing *ITM2B*, which is essential for *APP* metabolism and reducing amyloid plaques [39, 40]. Like *BLC6*, the MR-TF *MAFF* (MAF BZIP Transcription Factor F) is a repressor implicated in neurodegenerative diseases, including AD. *MAFF* acts as a binding partner of *NRF2* that regulates the cellular response to oxidative stress and inhibits inflammation. *MAFF* overexpression suppresses *NRF2* by forming homodimers that prevent NRF2 binding, thereby influencing AD pathogenesis by inhibiting *NRF2*-regulated genes and modulating the response to oxidative stress. Similarly, low *MAFF* expression silences transcription of *NRF2*-dependent genes, which may influence AD [41, 42].

Both *TEAD2* and *NFKBIA* regulate key signaling pathways governing neuronal survival, differentiation, and inflammatory responses. The TEA Domain Transcription Factor 2 (*TEAD2*) TF is a central downstream component of the Hippo pathway, implicated in promoting neuroinflammation, oxidative stress, and neuronal death when deregulated. *TEAD2* is considered a key factor in the development of AD, arising from mutations in the Hippo pathway and/or deregulation of YAP/TAZ (activation cofactors), which culminate in aberrant *TEAD* transcriptional activity and neuronal necrosis [43–45]. Besides, polymorphisms in the Nuclear factor κB (*NF-κB*) (*NFKBIA*) are associated with the pathogenesis of AD through mechanisms that involve neuronal survival, synaptic plasticity, microglial activity, and inflammation [46].

In relation to conservation and plasticity of regulatory networks, we identified MR-TFs with conserved regulons across regions, despite strong regional specificity. MR-TFs such as *MEF2C, RBFOX2*, and *GUCY1B1* maintained stable target gene sets across tissue pairs. These MR-TFs may support processes essential to neuronal homeostasis and neuronal development, whose disruption contributes to AD pathology [13, 47]. *MEF2C* is a myocyte enhancer factor expressed in the nervous system that acts as an immune checkpoint, limiting microglial overactivation, and is also associated with the risk of AD. Downregulation of *MEF2C* is associated with increased β-secretase (BACE1) activity, an enzyme that promotes Aβ production [13, 48].

*RBFOX2* (also known as *RBM9* and *FOX2*) is a member of the RBFOX family and regulates alternative exon splicing in the nervous system and other cell types, as well as cell proliferation and invasive migration [47, 49]. *RBFOX2* mutations are linked to intellectual disability and neurodevelopmental disorders. Although not directly associated with AD, *RBFOX2* regulates the alternative splicing of mRNAs (e.g., *MAPK* and *CYFIP2*) that are critical for proper neuronal function, and its deregulation is also related to neurodegenerative disorders and increased risk of AD [47, 50]. Furthermore, GUCY1B1 encodes the β subunit of soluble guanylate cyclase, a component of the NO–sGC–cGMP signaling pathway. Alterations in *GUCY1B1* may influence AD susceptibility by modulating Nitric oxide-dependent synaptic plasticity, oxidative stress, neuroinflammation, and myelination processes that are critical for cognitive function maintenance [51]**.**

The overlap between MR-TFs and susceptibility loci identified by GWAS reveals functional convergence between genetic predisposition and transcriptional deregulation. MR-TFs *SPI1, EGFR, and BIN1* reinforce the hypothesis that genetic variants associated with AD exert their effects by modulating regulon programs and pathways involved in brain homeostasis [1, 52]. A recent multi-ancestry GWAS highlights risk variants for AD in *SPI1, TREM2, PTK2B*, and *CR1*. Such MR-TFs and genes are associated with microglial expression and activity [1, 52, 53].

We identified MR-TFs related to the glial cell and inflammatory response core in AD. The *SPI1* (Spi-1 Proto-Oncogene) gene is a well-known master regulator of the inflammatory response in microglia and was identified, together with the *MEF2C* gene, as a key regulator of AD subtypes [13]. Bridging integrator 1 (*BIN1*) is expressed in microglia, oligodendrocytes, and neurons, and is responsible for regulating endocytosis, intracellular trafficking, and synapse function. Its expression levels are associated with AD pathology and influence the regulation of APP metabolism and Aβ production, as well as tau levels [54]. Thus, Epidermal growth factor receptor (*EGFR*) acts on neurogenesis and neuronal stem cell proliferation, and the recurrence of its expression levels is associated with inflammatory response, astrocyte activation, and induction of Aβ neurotoxicity [55, 56].

In overlap with DGE results, GWAS, and AD-PPI, we identified three genes associated with glial responses in AD pathology. Triggering receptor expressed on myeloid cells 2 (*TREM2*) is a key regulator of microglial function and inflammatory responses and is associated with an increased risk of late-onset AD [29, 57]. Complement receptor 1 (*CR1; CD35*), expressed on astrocytes and microglia, has a glial function in regulating the activation of the complement protein C3b [30]. The Protein tyrosine kinase 2β (*PTK2B*) gene, expressed in neurons and microglia, regulates microglial activity and biological processes related to plasticity, neuronal development, and the inflammatory response [28].

This convergence positions MR-TFs as key functional intermediates linking inherited genetic risk to AD-associated cellular states, providing a mechanistic framework for interpreting genetic associations within human brain biology. Integration of inferred MR-TF regulons with AD-PPI, GWAS signals, and TFLink revealed convergent regulatory and physical interactions supported by experimental validation. Notably, MR-TF-centered regulons with the strongest convergent support, including those centered on *CDK2*, *SPI1*, and *RBFOX2,* were experimentally evidenced by AD-PPI data and TFLink interactions.

## Cellular Dimension of Regulation in AD

Although based on bulk data, our analyses revealed cellular dimensions of regulatory dysfunction in AD. Regions such as the parahippocampal gyrus and temporal cortex exhibited a broader range of MR-TFs across cell types, including astrocytes, neurons, microglia, oligodendrocytes, and endothelial cells. This finding reflects the cellular complexity and heterogeneity of AD, which involves coordinated reprogramming of multiple cell types [58].

The recurrence of potential MR-TFs associated with astrocytes underscores their role in the mechanical mechanisms underlying AD pathology. At the same time, the presence of neuronal regulators points to the progressive loss of neuronal identity and plasticity as structural components of the disease [26]. Furthermore, neuroinflammation reinforces the central role of innate immunity in AD by engaging microglia and astrocytes in Aβ aggregate clearance mechanisms [59].

The MR-TF *SOX9* was mapped for astrocyte and neuronal cells. *SOX9* acts in nervous system development, particularly in the specification of astrocyte and oligodendrocyte cell fates [60]. *SOX9* and *ZFP36L1* were associated with astrocytes. In contrast, *SPI1* and *NFKB1A* were associated with microglial subpopulations as inflammatory and lipid-associated in a constructed cell atlas and in analyses across disorders, including AD [8, 58, 61–63]. Additionally, *SOX9* and *SPI1* were identified as MR-TFs in the overlap between mouse models and human AD [60]. Mathys et al. characterized cell-type diversity and identified four astrocyte subpopulations. Among the marker genes, the MR-TF *SOX9* emerges as a defining marker of astrocyte subpopulation 1 (Ast1) [8].

A similar network reconstruction analysis highlighted a TF-target regulatory network in which *SPI1* is a central gene associated with microglial status and AD-related inflammatory pathology [63, 64]. These findings reinforce the regulatory role of MR-TFs in AD, including the differentiation of neuronal and glial cells that modulate neurodegeneration and neuroinflammation.

This study is limited to using bulk RNA-seq, which does not allow direct association of regulatory effects from changes in cell composition. However, the consistency of results across multiple independent cohorts, combined with integration with genetic, TF-target interaction data and AD-PPI data, offers robustness to the conclusions. Future studies that integrate single-cell transcriptomics and regulatory epigenomics data will be essential for validating and dissecting the roles of regulons at the cellular level.

## Conclusion

Our results demonstrate that AD is underpinned by a hybrid regulatory architecture, in which a core of conserved transcriptional programs coexists with extensive regional and cellular plasticity. Through network reconstruction of TF-centered regulatory networks across brain tissues in AD, we identified novel MR-TFs, including *ADCYAP1, RBFOX2, MEF2C*, and *GUCY1B1*. These MR-TFs likely support processes essential to neuroinflammation and neurodegeneration, and are associated with astrocytes and microglia. In addition, we identified the MR-TFs *BIN1, SPI1,* and *EGFR,* which were identified in AD-related GWAS. Several MR-TF-target interactions were supported by TF-target and AD-PPI experimental data. This work provides an integrative framework for understanding how genetic risk, transcriptional deregulation, and cellular dysfunction converge in the pathogenesis of AD. Overall, these findings reveal the molecular architecture of AD from the perspective of master regulatory networks.

## Data Availability

The harmonized RNA-seq data for ROSMAP, MSBB, and Mayo cohorts are available at (https://www.adknowledgeportal.synapse.org/Explore/Studies/DetailsPage/StudyDetails?Study=syn21241740).
